# E2F1 Expression and Apoptosis Initiation in Crayfish and Rat Peripheral Neurons and Glial Cells after Axonal Injury

**DOI:** 10.3390/ijms23084451

**Published:** 2022-04-18

**Authors:** Valentina Dzreyan, Moez Eid, Stanislav Rodkin, Maria Pitinova, Svetlana Demyanenko

**Affiliations:** Laboratory of Molecular Neurobiology, Academy of Biology and Biotechnology, Southern Federal University, Stachki Ave., 194/1, 344090 Rostov-on-Don, Russia; dzreyan2016@mail.ru (V.D.); rodkin_stas@mail.ru (S.R.); maria.pitinova@mail.ru (M.P.); demyanenkosvetlana@gmail.com (S.D.)

**Keywords:** apoptosis, E2F1 inhibition, crayfish neuron, dorsal root ganglion, E2F1, nerve injury

## Abstract

Neurotrauma is among the main causes of human disability and mortality. The transcription factor E2F1 is one of the key proteins that determine the fate of cells. The involvement of E2F1 in the regulation of survival and death of peripheral nerve cells after axotomy has not been previously studied. We, for the first time, studied axotomy-induced changes in the expression and localization of E2F1 following axonal injury in rats and crayfish. Immunoblotting and immunofluorescence microscopy were used for the analysis of the expression and intracellular localization of E2F1 and its changes after axotomy. To evaluate whether this transcription factor promotes cell apoptosis, we examined the effect of pharmacological inhibition of E2F activity in axotomized rat models. In this work, axotomy caused increased expression of E2F1 as early as 4 h and even 1 h after axotomy of mechanoreceptor neurons and ganglia of crayfish ventral nerve cord (VNC), as well as rat dorsal root ganglia (DRG). The level of E2F1 expression increased both in the cytoplasm and the nuclei of neurons. Pharmacological inhibition of E2F demonstrated a pronounced neuroprotective activity against axotomized DRGs. E2F1 and downstream targets could be considered promising molecular targets for the development of potential neuroprotective agents.

## 1. Introduction

Peripheral nerve damage is a complex process, and future research is needed to understand its neuronal and molecular mechanisms [1,2,3]. There are no effective neuroprotectors for the protection of nerve cells in the first hours after injury [3]; therefore, studies of the molecular mechanisms of neurodegeneration and neuroprotection on various model objects of neurotrauma remain relevant [4].

Molecular-cellular responses to neurotrauma have common features across species. For example, in invertebrates and mammals, axotomy induces similar responses from neurons and glia [5,6]. Previously, ultrastructural changes were shown in axotomized hippocampal neurons [6,7,8] and rat dorsal root ganglia [6], as well as in invertebrates, including aplysia [9], earthworm, squid and crayfish [10,11]. In our laboratory, the electrophysiology, morphology, ultrastructure and biochemistry of mechanoreceptor neurons (MRN) of crayfish and surrounding glial cells were previously studied in detail, as well as their responses to various physicochemical effects, inhibitors, or activators of various proteins [12,13,14,15,16]. As a result of these experiments, our team demonstrated the involvement of a number of signaling pathways and transcription factors in the survival and death of neurons and glial cells during axotomy. For example, our early proteomic studies in invertebrates revealed the expression of more than 100 proteins, including the pro-apoptotic proteins p53, E2F1, and c-myc, in axotomized ganglia of crayfish [17], which prompted further study of this transcription factor in the context of axonal injury.

It has been suggested that the survival and death of neurons and glial cells are controlled by certain signaling processes and transcription factors [4,18]. One of the key proteins that determine the fate of cells is the transcription factor E2F1 [19,20,21,22]. It controls the expression of genes that regulate DNA repair [22,23,24,25,26,27], proliferation, and apoptosis [28,29]. E2F1 predominantly binds to the promoter regions of cell cycle-related genes in primary cortical neurons, and its expression triggers cell cycle activation and neuronal apoptosis [22]. E2F1 engages cell-death pathways either alone or in cooperation with p53 to protect organisms from the development of tumors [28]. Furthermore, E2F1 participates in the development and the differentiation of several tissues involved in global metabolic homeostasis [19]. In turn, its expression is controlled by the p38 MAP kinase and the c-Myc transcription factor [19,21].

In the cell nucleus, E2F1 as a transcription factor controls the expression of various proteins that regulate many processes, including apoptosis [21]; however, in the cytoplasm, E2F1 can act in a non-transcriptional way [25,30], directly interacting with mitochondria [31,32]. All of this makes this protein an interesting object for study. Only a few studies have demonstrated the involvement of this protein in traumatic injury to peripheral nerves [33,34]. The involvement of E2F1 in the regulation of survival and death of neurons and glial cells during axotomy has not been previously studied.

This work continues a series of studies on the evolutionary aspects of neurotrauma in invertebrates and mammals. Conducting experiments on several experimental models at once, both on vertebrates and invertebrates makes it possible to obtain comparative data that can serve as a theoretical basis in order to better understand the fundamental mechanisms of survival and death of neurons and glial cells in the case of peripheral nerve damage (axotomy). Studies of nerves in invertebrates are also justified by the fact that in our previous studies, during proteomic analysis and immunoblotting, an increase in the level of E2F1 in axotomized ganglia of both mammals and crayfish *Astacus Leptodactylus* was noted as early as 1 h after axonal damage [17,34]. As a result, we concluded that E2F1 is conservative in nature, which was also confirmed by other studies on various model organisms of different levels of organization [28,35].

We studied axotomy-induced changes in the expression and localization of E2F1 in mechanoreceptor neurons (MRN) and ganglia of the crayfish ventral nerve cord (VNC), as well as in the rat dorsal root ganglia (DRG). As a result of the work, we have shown for the first time the intracellular localization of E2F1, its changes after axotomy, and also determined in which cells, neurons, or glia, this protein is expressed. To evaluate whether this transcription factor promotes cell apoptosis in axotomized rat spinal ganglia, we examined the effect of pharmacological inhibition of E2F activity in axotomized rat models. Importantly, our results indicate that E2F1 is critical for the induction of apoptosis, and downregulation of E2F1 activity may have a protective role against axotomy-induced cell death of rat DRG.

## 2. Results

### 2.1. Expression of E2F1 in Axotomized Rat DRG and Ganglia of Crayfish VNC and Their Nuclear and Cytoplasmic Fractions

**Figure 1:**
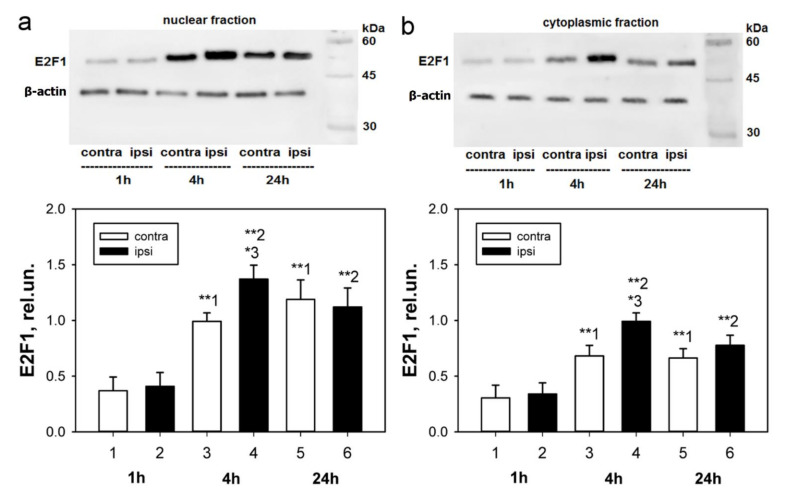
Immunoblotting. Changes in E2F1 level in nuclear (**a**) and cytoplasmic (**b**) fractions of axotomized ipsilateral DRG in 1, 4 and 24 h after sciatic nerve transection in rats in comparison with contralateral ganglia of the same animals. Denotation: rel.un—the ratio of the optical density of the strip of the studied protein to the optical density of the strip of the protein load marker (actin), ipsi—axotomized ipsilateral ganglion, contra—contralateral control ganglion. The columns are numbered for comparison. Two Way ANOVA. M ± SEM. n = 7. * *p* < 0.05; ** *p* < 0.01.

Unilateral transection of the right sciatic nerve caused an increase in the level of the E2F1 protein relative to the intact ganglia both in the cytoplasmic and nuclear fractions of rats DRG 4 h after transection: by 43% (*p* < 0.05) and 40% (*p* < 0.05), respectively (Figure 1). Immunoblotting showed that in both axotomized and control intact DRG, protein expression significantly increased at 4 and 24 h compared to the level determined 1 h after transection of the sciatic nerve (*p* < 0.01), which reflects a nonspecific response of the rat’s nervous system for damage (Figure 1). At the same time, the increase in protein in the nuclear fraction during the first 24 h after injury was more pronounced (Figure 1a).

**Figure 2:**
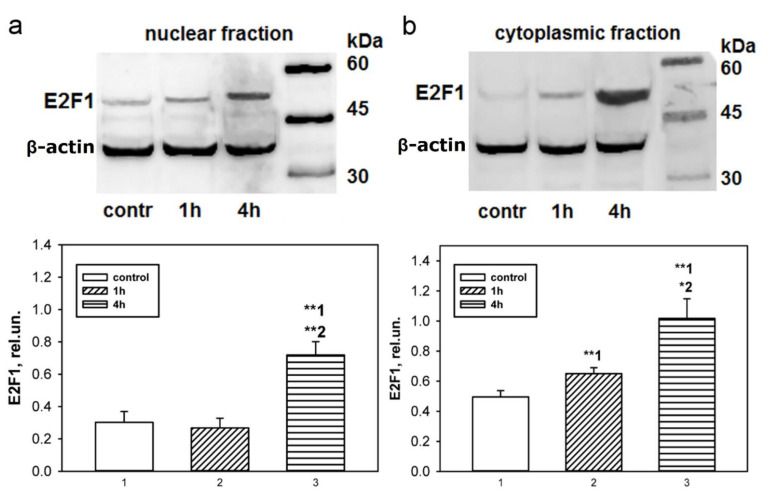
Immunoblotting. Changes in E2F1 level in nuclear (**a**) and cytoplasmic (**b**) fractions of bilaterally axotomized ventral nerve cord ganglia of crayfish in 1 and 4 h after the transection of interganglionic connectives: Denotation: rel.un.—the ratio of the optical density of the strip of the studied protein to the optical density of the strip of the protein load marker (actin), control—intact control. The columns are numbered for comparison. One Way ANOVA. M ± SEM. n = 7. * *p* < 0.05; ** *p* < 0.01.

According to immunoblotting data, the baseline level of E2F1 in the control ganglia of the crayfish ventral nerve cord was low (Figure 2). One hour after bilateral axotomy of the VNC ganglia, a significant increase in protein expression by 40% was observed in the cytoplasmic fraction of the VNC ganglia (*p* < 0.01, Figure 2b), although its level in the nuclear fraction did not change (Figure 2a). As can be seen in Figure 2b, after 4 h the increase in E2F1 in the cytoplasmic fraction became more significant: 2 times compared with the control value (*p* < 0.01) and 1.5 relative to the protein level after 1 h (*p* < 0.05). The E2F1 levels in the nuclear fraction 4 h after axotomy increased almost 2.5 times relative to the control values (*p* < 0.01) and relative to the protein level after 1 h (*p* < 0.01, Figure 2a).

### 2.2. Localization of E2F1 in the Rat DRG Neurons 1, 4, 24 h, and 7 Days after Transection of the Sciatic Nerve

**Figure 3:**
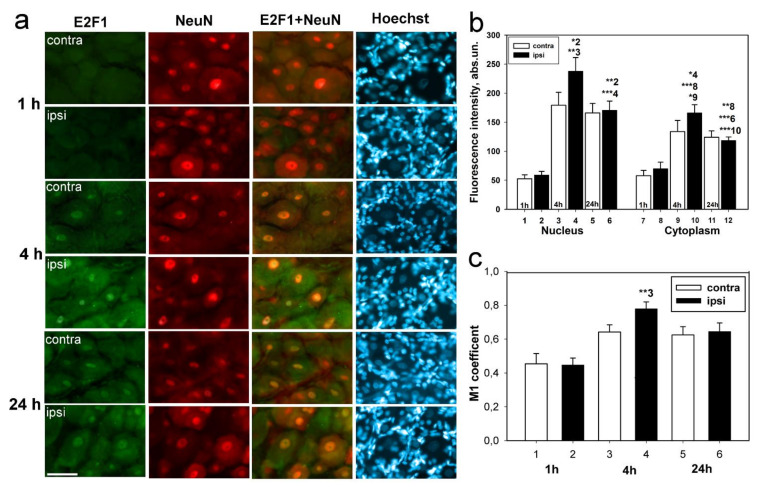
Fluorescence microscopy. (**a**) Expression of E2F1 (green fluorescence) in rat DRG neurons in 1, 4, and 24 h after sciatic nerve transection. Scale bar 50 µm. (**b**) Fluorescence intensity of anti-E2F1 antibody in nuclei and cytoplasm of neurons in axotomized ipsilateral and control contralateral DRG in 1, 4, and 24 h after sciatic nerve transection. (**c**) Colocalization coefficient M1 of E2F1 and neuronal nuclear marker in axotomized ipsilateral and control contralateral DRG in 1, 4, and 24 h after sciatic nerve transection. Denotation: ipsi—axotomized ipsilateral ganglion, contra—contralateral control ganglion. NeuN—neuronal nuclear marker; E2F1 + NeuN—the overlapping of E2F1 antibody fluorescence and NeuN fluorescence. Hoechst—Hoechst 33342 fluorescence, imaging all neuronal and glial cell nuclei. The columns are numbered for comparison. Two Way ANOVA. M ± SEM. n = 7. * *p* < 0.05; ** *p* < 0.01; *** *p* < 0.001.

**Figure 4:**
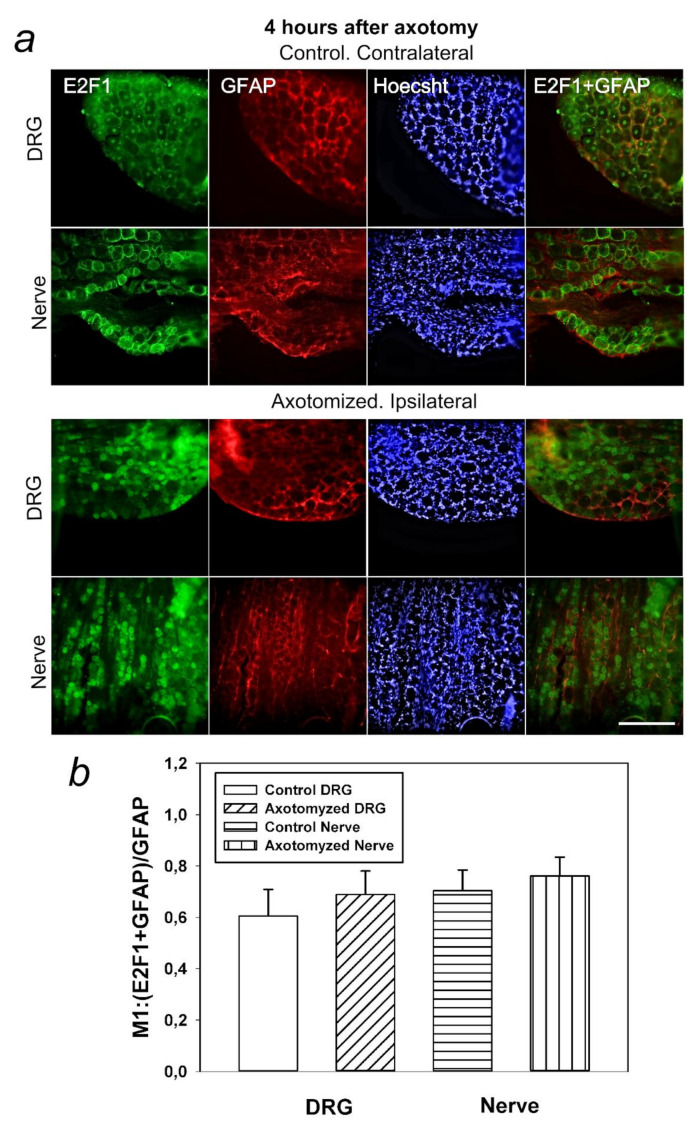
Colocalization of E2F1 with the glial marker GFAP (glial fibrillary acidic protein) and the nuclear chromatin marker Hoechst 33342 in the DRG at 4 h after unilateral transection of the right sciatic nerve compared to control ganglia of the contralateral intact side of the same animal. (**a**) Immunofluorescence of E2F1 (green), GFAP (red), or Hoechst 33342 (blue), and merged images. Scale bar 100 μm. (**b**) Colocalization coefficient M1 of E2F1 and GFAP in axotomized ipsilateral and control contralateral DRG in 4 h after sciatic nerve transection. Student’s *t*-test. M ± SEM. n = 7.

The results of immunoblotting were confirmed by immunofluorescence microscopy (Figure 3 and Figure 4). Large DRG neurons expressing E2F1 are surrounded by many glial cells, whose nuclei are detected by the Hoechst 33342 fluorochrome (Figure 3a). E2F1 was localized both in DRG neurons and the glial cells (Figure 3a and Figure 4a), as evidenced by the low coefficient of E2F1 colocalization with the glial marker GFAP (glial fibrillary acidic protein) (Figure 4b).

E2F1 was practically absent in DRG 1 h after transection of the sciatic nerve. But 4 h after axotomy, the average level of E2F1 in axotomized ganglia significantly increased relative to the 1-h group both in the cytoplasm of neurons (2.5 times, *p* < 0.001), and especially in their nuclei (almost 5 times, *p* < 0.05), where the E2F1 fluorescence was at maximum (Figure 3b). The increased localization of E2F1 in the karyoplasm (nucleoplasm) of DRG neurons is evidenced by an increase in the Manders’ coefficient of colocalization of E2F1 with the neuronal marker NeuN (Figure 3c). The E2F1 level in axotomized DRG after 4 h increased in the cytoplasm of neurons by 15% (*p* < 0.05) and in the nuclei by 30% (*p* < 0.01), respectively. Increased expression of E2F1 in axotomized ganglia 4 h after transection of the sciatic nerve is associated with increased protein expression in neurons, but not in DRG glial cells: the Manders’ coefficient of colocalization of E2F1 with the GFAP, which stains mainly the glial cells (Figure 4b) did not change compared with intact ganglia.

24 h after transection of the sciatic nerve, the level of E2F1 immunofluorescence relative to the contralateral ganglia did not change, but there was a decrease in the intensity of E2F1 fluorescence in the nuclei and cytoplasm of neurons in both axotomized and control ganglia relative to the 4-h group (Figure 3b).

### 2.3. Axotomy-Induced Apoptosis in the DRG and Nerve of the Rat 7 Days after Unilateral Transection of the Right Sciatic Nerve

Figure 1, Figure 3 and Figure 4 show that E2F1 increased expression occurs simultaneously in most DRG cells. This is characteristic of both the cytoplasm of cells and their nucleus. Moreover, the majority of DRG neurons were found to be E2F1 positive; however, not all neurons die when they are axotomized. In this sense, to clarify the role of E2F1 in DRG cell death, we estimated the percentage of E2F1-positive and negative neurons that died after axotomy using TUNEL assay.

Visualization of apoptotic cells was performed using the TUNEL assay (Terminal deoxynucleotidyltransferase (TdT)-mediated dUTP nick end labeling), which marks DNA strand breaks using the In Situ Cell Death Detection Kit. In our previous studies, it was shown that transection of the rat sciatic nerve induced apoptosis of DRG glial cells remote from the transection site 24 h after axotomy, while apoptosis of DRG neurons began 7 days later. Therefore, in this work, the results of visualization of apoptotic cells were presented only 7 days after transection of the sciatic nerve, since we can observe apoptosis of both glia and neurons by this time.

**Figure 5:**
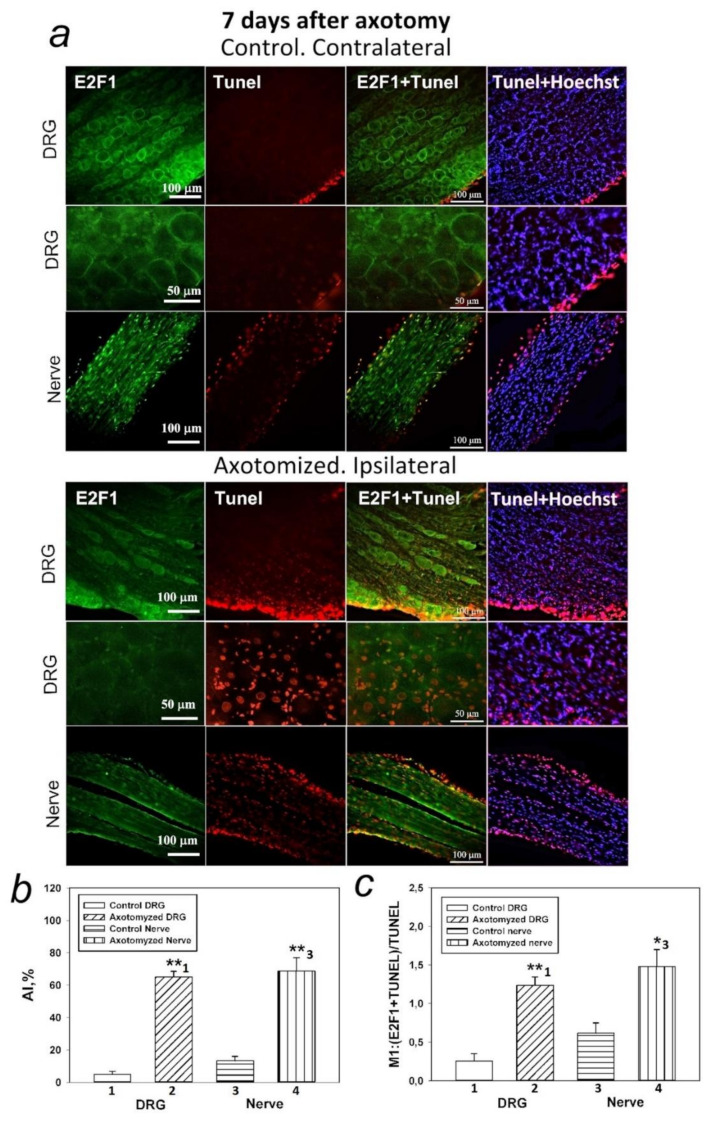
Axotomy-induced apoptosis in the DRG and nerve of the rat. (**a**) Typical images of the rat DRG and nerves stained with anti-E2F1 (green), and visualization of apoptosis by TUNEL (red) in control and axotomized groups at 7 days after axotomy. Scale bar 100 μm and 50 μm. (**b**) The changes in the apoptotic index (AI, %) in control and axotomized groups. (**c**) Manders’ coefficient M1 displays the axotomy-induced co-localization of E2F1 with TUNEL-positive nuclei. The columns are numbered for comparison. Student’s *t*-test. M  ±  SEM. n  =  7. * *p*  <  0.05 relative to the control groups, ** *p*  <  0.01 relative to the control groups.

As shown in Figure 5, the percentage of apoptotic cells in axotomized DRGs increased significantly (10–13 times compared with intact ganglia) 7 days after unilateral transection of the right sciatic nerve in both neurons and glial cells (Figure 5b). The increased colocalization of E2F1-positive cells with the apoptosis marker TUNEL (Figure 5c), compared with control contralateral DRG, suggested that apoptosis in axotomized ganglia was associated with increased expression of E2F1.

### 2.4. Localization of E2F1 in the Crayfish Mechanoreceptor Neuron 4 and 8 h after Axotomy

**Figure 6:**
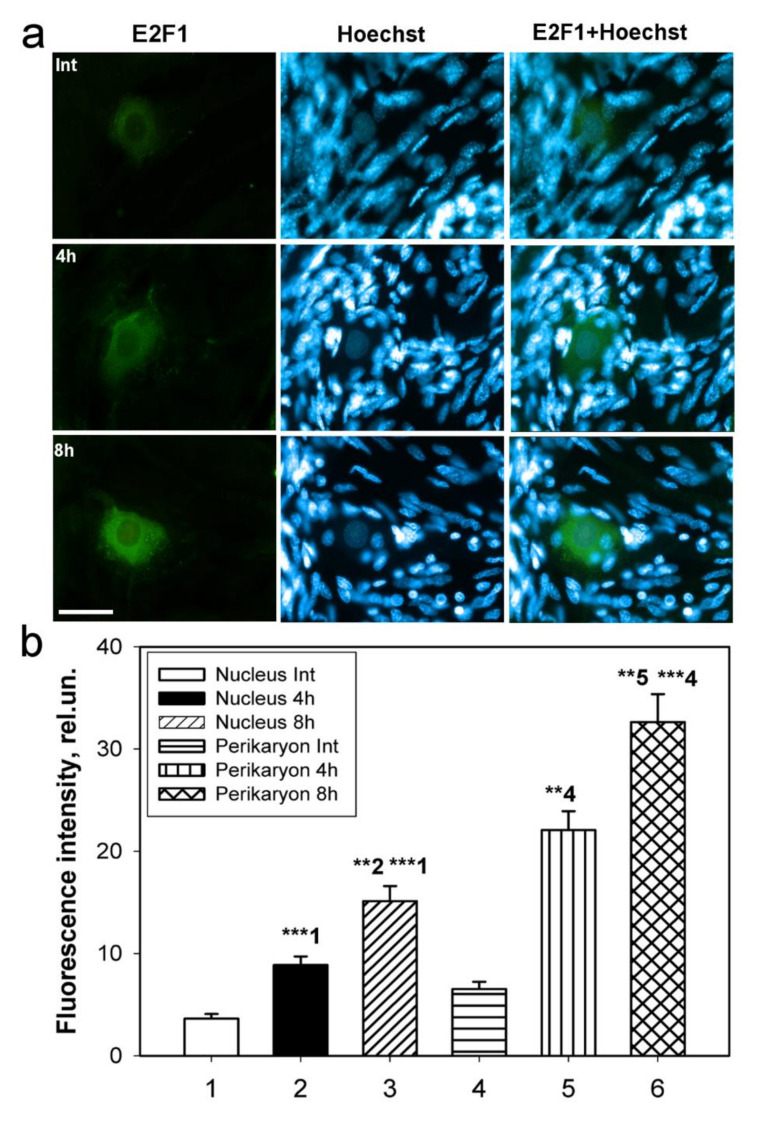
Fluorescence microscopy. Intracellular distribution of E2F1 (**a**) in crayfish stretch receptor in 4 and 8 h post-axotomy and its colocalization with nuclear marker Hoechst 33342. Scale bar 100 µm. Fluorescence of anti-E2F1 (**b**) antibody in different parts of stretch receptor neuron: nucleus, perikaryon, axons, and dendrite of intact neurons (Int), connected with ventral nerve cord ganglia and in axotomized neurons in 4 h and 8 h after axon transection. Rel.un.—the unit after estimating the average fluorescence (by area) of the nucleus and perikaryon, subtracting the background and dividing by the background. The columns are numbered for comparison. One Way ANOVA. M ± SEM. n = 10. ** *p* < 0.01; *** *p* < 0.001.

More detailed changes at the cellular level were observed at the isolated crayfish stretch receptor (Figure 6). The micrographs clearly showed that E2F1 was localized only in the MRN body, but not in the axon and dendrites and not in the surrounding glial cells (Figure 6a). In control intact MRNs, in which the axon was not damaged and retained its connection with the corresponding VNC ganglion, E2F1 expression was practically not observed (Figure 6a,b). The E2F1 level increased sharply in the perikaryon 4 h after axotomy (*p* < 0.01) and even more after 8 h (*p* < 0.001). The intensity of E2F1 fluorescence increased not only in the perikaryon but also in the nucleus, although a bit weaker. Thus, 4 h after cutting the axon in the MRN, the level of E2F1 in comparison with the control group increased 2 times in the nucleus (*p* < 0.001), and almost 3 times in the perikaryon (*p* < 0.01). In 8-h axotomized MRNs, E2F1 fluorescence increased relative to intact neurons in the nucleus by 3.4 times (*p* < 0.001) and in the perikaryon by 4.4 times (*p* < 0.001), and relative to neurons incubated for 4 h after axotomy, by 1.7 (*p* < 0.01) and 1.6 (*p* < 0.01) times, respectively (Figure 6b).

### 2.5. Influence of Pharmacological Inhibition of E2F1 In Vivo on the Level of Apoptosis and the Content of Proapoptotic Proteins in Axotomized Rat DRGs

To evaluate the effect of pharmacological inhibition of E2F1 in vivo on the level of apoptosis and the content of proapoptotic proteins in axotomized rat DRGs, we selected the E2F chemical inhibitor HLM006474, abbreviated here to 6474 (Sigma-Aldrich). The intraperitoneal administration of this inhibitor to rats was for 7 days.

**Figure 7:**
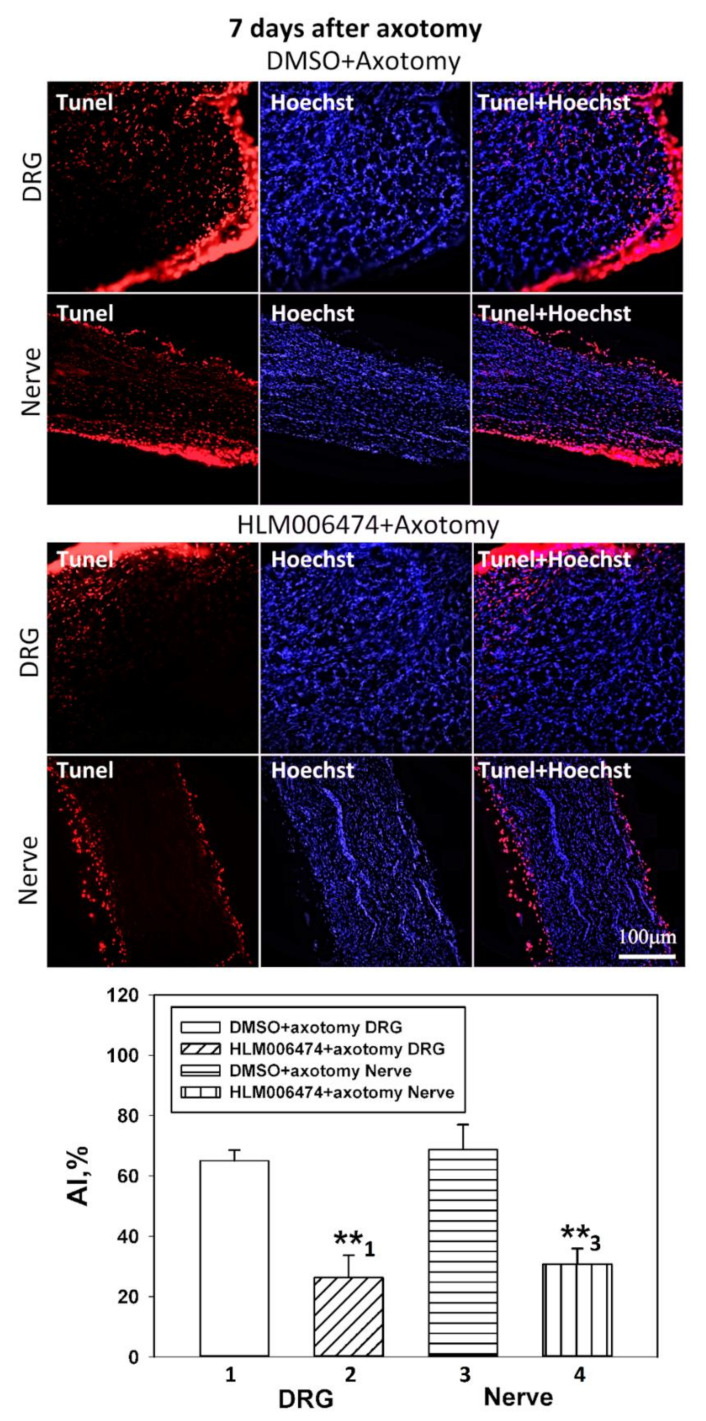
The effect of E2F chemical inhibitor HLM006474 on the apoptotic index (%) both in the damaged ipsilateral DRG and in the damaged ipsilateral nerve (HLM006474 + axotomy) compared with the control group. Rats were injected daily with DMSO, or HLM006474, for 7 days after axotomy (DMSO + axotomy). The columns are numbered for comparison. Student’s *t*-test. M ± SEM; ** *p* < 0.01.

**Figure 8:**
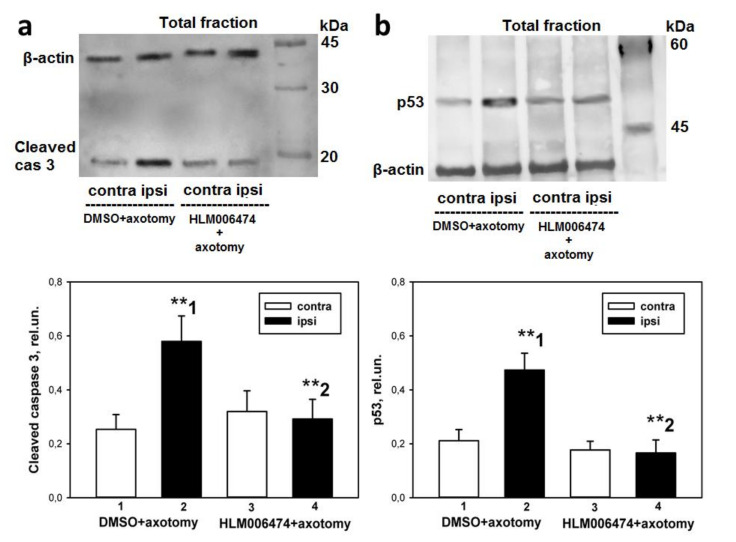
The effect of E2F chemical inhibitor HLM006474 on the level of cleaved caspase 3 (**a**) and p53 (**b**) in the total fraction of the axotomized right dorsal root ganglion (ipsi) as compared with the undamaged contralateral (left) ganglion (contra) at 7 days after axotomy (AT). The columns are numbered for comparison. Two Way ANOVA. M ± SEM; ** *p*  <  0.01.

The administration of 6474 significantly reduced apoptosis (the apoptotic index, %) both in the damaged ipsilateral DRG and in the damaged ipsilateral nerve compared with the control group (which were injected with DMSO), by 1.6 (*p* < 0.01) and 1.7 (*p* < 0.01) times, respectively (Figure 7). Increased expression of cleaved caspase 3 and transcription factor p53 in rat DRG after neurotrauma was demonstrated by us earlier [34]. The earliest proapoptotic event in the injured DRG was increased expression of transcription factor E2F1 at 4 h after sciatic nerve transection. This preceded the induction of p53 and cleaved caspase 3 at 24 h post-axotomy. Administration of 6474 significantly reduced the expression of cleaved caspase 3 (*p* < 0.05, Figure 8a) and transcription factor p53 (*p* < 0.01, Figure 8b) caused by SNT in the total fraction of the DRG.

Thus, pharmacological inhibition of E2F demonstrated a pronounced neuroprotective activity against axotomized DRGs.

## 3. Discussion

The transcription factor E2F1 plays an important role in the regulation of critical cellular processes, including cell cycle arrest and apoptosis [19,21,28,36], which makes it the object of intensive research in the context of cancer prevention and treatment [20,28]. However, in addition to the role of E2F1 in tumor defense mechanisms, there is evidence to date that E2F1 mediates pathological cell death that causes tissue destruction (e.g., neuronal degeneration and ischemic cell damage) [17,36,37,38]. Recent studies on various models of CNS damage, including spinal trauma [32,33,39], neurodegenerative pathologies [40,41], and cerebral ischemia [42], have revealed an increased expression of this protein. These results once again prove that E2F1 may play a key role in diseases affecting the nervous system.

Our experiments showed an increased expression of E2F1 in various model objects of invertebrates and vertebrates: in the bilaterally axotomized ganglia of the crayfish VNC, in the nuclei and cytoplasm of the mechanoreceptor neuron, as well as in the axotomized dorsal root ganglia of rats in the early stages after axotomy. This made it possible to study in detail the intracellular localization and expression of E2F1 both in the neurons of vertebrates, using the rat as an example, and in the nerve cells of invertebrates, such as the crayfish *Astacus Leptodactylus*. Thus, the rabbit antibodies against mammalian E2F1 used in this work perfectly recognized the corresponding epitopes in the homologous crayfish protein. This indicates the conservatism of the E2F1 protein and its presence in the nervous system of invertebrates, including crustaceans [28,35].

Immunoblotting and immunofluorescence microscopy revealed increased expression of E2F1 as early as 4 h and even 1 h after axotomy of mechanoreceptor neurons and ganglia of crayfish VNC, as well as rat DRG. These results are consistent with the data of previous experiments with proteomic microarrays, in which a 1.8-fold increase in E2F1 expression in axotomized ganglia of crayfish VNC was observed as early as 1 and 3 h after axotomy [17]. It can be seen that E2F1 expression in axotomized ganglia and MRN reaches its maximum by 4 h after axotomy and gradually decreases thereafter. This suggests an early E2F1-dependent response in neurons that develops under stress. It is important to note here the fact that 7 days after transection of the sciatic nerve, the expression of E2F1 has significantly decreased. Moreover, this was observed both in the damaged ganglia and in the control. These results also support the fact that E2F1 may be directly involved only in the initiation of apoptosis, after which its content in the cell tends to a minimum.

We have previously shown an increase in the E2F1 expression of the total fraction of axotomized rat DRG 4 h after transection of the sciatic nerve [36]. Considering the fact that E2F1 can also act in a non-transcriptional way in the cytoplasm of cells [25], we were interested in evaluating the changes in the level of this protein separately in the nuclear and cytoplasmic fractions of axotomized ganglia 1, 4, and 24 h after axotomy.

An important observation was that the level of E2F1 expression in our experiments increased both in the cytoplasm and the nuclei of neurons, as evidenced by both immunoblotting and immunofluorescence microscopy. In the case of cytoplasmic localization, one of the possible functions of E2F1 is its interaction with mitochondria and regulation of their functions, for example, by direct interaction with the Bcl-xL protein on the outer mitochondrial membrane and regulation of its permeabilization [31,32]. To unravel the mystery of the functions of cytoplasmic E2F1 during axotomy, additional experiments planned by us in the near future will be needed. In these future experiments, we will try to consider the causal relationship of E2F1 translocation from the nucleus to the cytoplasm after axotomy and exclude the option of random localization of E2F1 in the cytoplasm.

In the nucleus, E2F1 apparently acts as a transcription factor. It is known that it transcriptionally induces the expression of pro-apoptotic proteins, such as caspases 3, 7, 8, and 9, SMAC/DIABLO, Apaf-1, p53, p73, proteins of the Bcl-2 family and thereby stimulates apoptosis [19,21,28,29,36]. As shown earlier in a proteomic study, in axotomized ganglia of crayfish VNC, the levels of these pro-apoptotic proteins increased simultaneously with E2F1 [17]. This suggests a possible role of E2F1 in neuronal and glia apoptosis. Having obtained data indicating an association between increased expression of E2F1 and cell death of axotomized DRGs, our next step was to develop a hypothetical mechanism explaining exactly how E2F1 is involved in nerve damage and the death of neurons and glial cells.

The obtained results prompted us to pharmacologically intervene in the E2F axis. Four E2F inhibitors have been described, three of which are peptides [43] and one is a small molecule inhibitor (HLM006474, abbreviated here to 6474) [44,45,46,47]. To the best of our knowledge, none of them has been tested in vivo or used in cases of peripheral nerve injury. Intraperitoneal administration of this inhibitor to rats for 7 days completely abolished the axotomy-induced increased expression of proapoptotic active caspase 3 and p53 proteins, and protected axotomized DRG cells from apoptosis as well. Therefore, the transcription factor E2F1 may be involved in axotomy-induced damage to DRG neurons and glial cells.

E2F inhibitor 6474 blocks the transcriptional activity of E2F by disrupting E2F–DNA binding [44]. Yihong Ma et al. have shown in several cell lines that overnight exposure to HLM006474 leads to suppression of known E2F targets, including the p53 transcription factor [48]. Therefore, the decrease in the level of transcription factor p53 in axotomized DRGs after 7 days of administration of 6474 to animals resembles the effect of reduced E2F activity and is direct evidence of the inhibitor’s effectiveness. Together, these results suggest that inhibitor 6474 has an effect in vivo, and interference with E2F activity using this compound may have clinical applications in the treatment of neurotrauma and its consequences.

Dysregulation of E2F1 expression induces apoptosis through p53-dependent and p53-independent pathways [28]. Literature data on the interaction of p53 and E2F1 remain rather controversial [28,32,41,49]. E2F1 directly binds both Mdm2 and p53, increasing the expression of the latter [50,51]. It has been shown that in cancer cells, p53 activation inhibits E2F1 activity, causing cell cycle arrest, or p53 interacts directly with E2F1 and triggers apoptosis [52]. On the contrary, in the case of nerve damage, the expression of E2F1 is increased, which stimulates the release of cytochrome C from mitochondria and ultimately leads to apoptosis [53]. Several studies have shown that E2F1 interacts with p53 by activating the expression of pro-apoptotic cofactors of p53, such as JMY and TP53INP1, as well as proteins that stimulate apoptosis (ASPP) ASPP-1 and ASPP-2 via a direct transcription mechanism [28]. From another study [47], it is known that the induction of apoptosis by E2F1 is mediated at least in part by the activation of p19ARF transcription, which inactivates MDM2, thereby stabilizing p53. Ma et al. observed the opposite course of pro-apoptotic phenomena: increased expression of p53 1 h after injury of the spinal cord was accompanied by increased expression of E2F1 3 h later [32]. Such contradictory data indicate that the mechanisms of apoptosis development, accompanied by the activation of these two proteins, remain incompletely understood.

There are no data on the relationship between p53 and E2F1 in peripheral nerve damage. This prompted us to the second important observation made as a result of the research: increased expression of E2F1 4 h after injury is accompanied by increased expression of p53, as well as activation of caspase 3, followed by the development of glial apoptosis 24 h later and the development of neuronal apoptosis 7 days after axotomy of the sciatic nerve of the rat. However, while caspase-3 promotes cellular degradation in the latter stages of the apoptosis pathway, caspase inhibitors do not always provide neuroprotection. This is due to the existence of caspase-independent pathways for apoptosis or other cysteine proteases, such as calpains or cathepsins, which are also involved in apoptotic neuronal death. Therefore, caspase inhibition is not a suitable strategy to prevent neurodegenerative processes.

A consistent increase in the expression of E2F1 and p53 was also shown by us through proteomic analysis of crayfish axotomized ganglia [17]. We suggest that, under axotomy conditions, the transcription factor E2F1 may be involved in the activation of p53 as a downstream target factor, which, in turn, causes secondary changes in the expression of genes and proteins that trigger apoptosis. Thus, increased expression of E2F1 may be a key event in the initiation of apoptosis of neurons and distant glial cells in axotomized DRG and prepares subsequent changes in other proteins, in particular p53, and the general response of ganglion cells in response to damage. For example, Camins et al. showed that inhibition of the E2F1/p53 pathway prevents neuronal apoptosis [49].

Our earlier in vivo inhibitory analysis on CSR neurons using p53 activators and inhibitors showed that axotomy-induced death of neurons and glial cells was associated both with the effect of p53 on transcription processes and with the transcription-independent effect of p53 on mitochondria in the cell cytoplasm [54]. These results, combined with the data of immunoblotting and immunofluorescence microscopy regarding nuclear and cytoplasmic localization of E2F1, suggest that the role of this protein in the death of nerve cells is not limited to its action as a transcription factor, but is also associated with its direct interaction with mitochondria and the p53 protein in the cytoplasm of cells. It is important to note that glial cells located at a great distance of several centimeters from the dissection site were more vulnerable to axotomy than DRG neurons. In fact, glial apoptosis was observed 24 h after SNT and intensified on day 7, while apoptosis of some DRG neurons did not begin until day 7 [36].

In this regard, it was also important for us to determine in which cells, neurons or glia, this protein is expressed. It was interesting to note that E2F1 was localized both in DRG neurons and the glial cells. However, the Manders’ coefficient of colocalization of E2F1 with the GFAP, which stains mainly the glial cells, did not significantly change compared to intact ganglia. Thus, increased expression of E2F1 in axotomized ganglia is associated with increased protein expression in neurons, but not in DRG glial cells. Furthermore, in the crayfish stretch receptor, E2F1 was expressed exclusively in neurons, but not in glial cells. It is possible that the features of E2F1 distribution can explain the different reactions of neurons and glial cells in response to axon transection.

Our data provide the first successful use of the E2F inhibitor HLM006474 in vivo, and its neuroprotective efficacy suggests that E2F1 is an important therapeutic target, because the performed inhibitory analysis demonstrated the involvement of the E2F1 transcription factor in triggering damage to DRG neurons and glial cells during axotomy. Apparently, the fate of neurons and glial cells in the axotomized rat ganglia and the crayfish stretch receptor is determined by the balance between different modalities of E2F1 activity. This will be the subject of our future research.

A detailed analysis of the disruption of the axotomized E2F1/p53 pathway is important for understanding the induction and development of neurodegeneration. The discovery of E2F1- and p53-independent pathways of apoptosis in neurons and glia of axotomized ganglia may identify new targets for the treatment of neurotrauma and its consequences. These considerations also will be the subject of our next work. Such a strategy will be aimed at saving cells from death and having a therapeutic effect, for example, restoring the number of neurons after neurotrauma as a conceptual alternative to cell transplantation.

E2F1 and p53 can be considered potential targets for the therapy of nerve damage, and their inhibitors should be studied as promising neuroprotective drugs.

## 4. Materials and Methods

### 4.1. Reagents and Antibodies

Xyla (2% xylazine hydrochloride solution) was purchased from Interchemie Werken “de Adelaar” BV (The Netherlands). Telazol (a combination of tiletamine and zolazepam hydrochlorides) was purchased from Zoetis (Kalamazoo, United States). Low-melting agarose was purchased from Dia-m Company (Moscow, Russia) (Agarose LM, Dia-m, 1925, 0025).

For the detection of E2F1 by immunofluorescence microscopy and immunoblotting, we used the anti-rabbit E2F1 (SAB2103144) antibody that recognizes the N-terminal domain of E2F1. We used the antibody anti- E2F1 (SAB2103144) according to the Producer‘s recommendations: https://www.sigmaaldrich.com/RU/en/product/sigma/sab2103144 (accessed on 4 October 2021), which confirms its applications for immunohistochemistry and western blot. The producer instruction states that anti-E2F1 (N-terminal) specifically recognizes E2F1 (N-terminal) by immunoblotting and immunoprecipitation (47 kDa). The antibody is also useful for the detection of E2F1 by immunohistochemistry. The epitope(s) recognized by the antibody is resistant to routine formalin-fixation and paraffin-embedding unless there is protease digestion. The negative control in our experiments—omission of the primary antibodies—confirmed the specificity of these antibodies. Another way to validate the specificity is by testing via protein microarray technology. Previously, we used this antibody in the protein microarray study of the rat brain and axotomized invertebrate ganglia [17,37]. It was a valid proof of its specificity.

All antibodies and other reagents used in this work were purchased from “Sigma-Aldrich-Rus” company (Moscow, Russia).

### 4.2. Animal Models

Axotomized mechanoreceptor neurons (MRN) and ventral nerve cord ganglia (VNC) were obtained from crayfish *Astacus Leptodactylus* purchased from a crayfish nursery.

**Figure 9:**
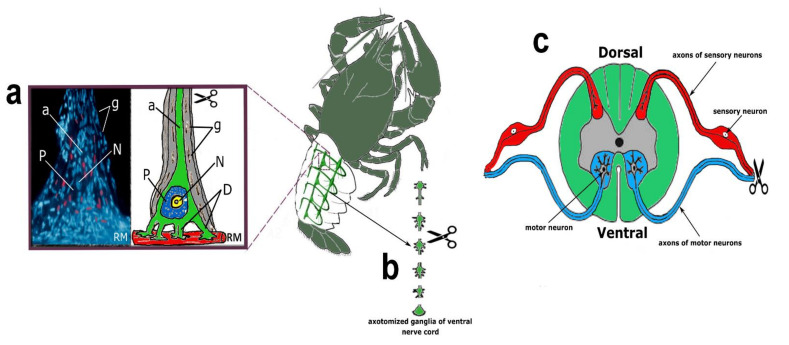
Experimental models of axotomy. (**a**)—stretch receptor neuron (SRN), stained with Hoechst 33342, which imparts blue fluorescence to nuclear chromatin, and propidium iodide, staining necrotic cell nuclei in red. The schematic picture of SRN morphology: N—nucleus, P—perikaryon, D—dendrites, g—glial cells, a—axon, RM—receptor muscle. (**b**)—axotomized ventral nerve cord ganglia. (**c**)—dorsal root ganglia of rat.

Experiments with transection of the sciatic nerve were carried out on adult male Wistar rats (200–250 g), which were kept in standard cages in groups of 4–5 animals with free access to food and water. Standard conditions were maintained in the animal holding room: 12h light/12h dark schedule, room temperature 22–25 °C, and air exchange rate 18 changes per hour. International, national, and/or institutional guidelines for the care and use of animals have been followed. All experimental procedures were carried out following European Union directives 86/609/EEC on the use of experimental animals and local legislation on the ethics of animal experiments. The animal protocols have been evaluated and approved by the Animal Care and Use Committee of the Southern Federal University (Permit No. 08/2016). Our study did not involve any human subjects. Experimental models of axotomy are shown in Figure 9.

#### 4.2.1. Crayfish Stretch Receptor (CSR)

CSR is a suitable model for studying the molecular mechanisms of neuronal and glial responses to axotomy. The mechanoreceptor neuron (MRN) is surrounded by a multilayer glial membrane. The structure of the MRN is shown in Figure 1a. Satellite glial cells (SGC) and their interaction with MRN were studied in detail at the optical and ultrastructural levels [12,13,14,15,16]. MRN generates impulses directed to the VNC ganglion with a frequency proportional to the lengthening of the receptor muscle, which is strengthened. MRN axotomy was performed by cutting the axon at a distance of 5–8 mm from the cell body. We have developed a technique for isolating control MRNs, the axon of which is not cut and retains a connection with the VNC ganglion [14]. The technique of MRN isolation and axotomy is described in more detail in our previous works [14,54].

#### 4.2.2. Crayfish Ventral Nerve Cord (VNC)

Axotomized ganglia of the crayfish ventral nerve cord consist of 6 ganglia containing 500–1000 neurons, interconnected by connectives consisting of several hundred axons. The structure of the axotomized ganglia of crayfish VNC is shown in Figure 1b. After chitin was removed from the ventral side of the crayfish tail, the VNC was quickly removed and transferred to a chamber with van Harreveld’s solution. The control VNCs were cut at the anterior and posterior ends, and in the experimental ones, the connectives were cut along the edges and between the ganglia so that six bilaterally axotomized ganglia were obtained [17,54].

#### 4.2.3. Rat Dorsal Root Ganglia (DRG)

Experiments on studying the level of E2F1 in the dorsal root ganglia after axotomy were carried out on rats, on which the technique of sciatic nerve transection (SNT) has already been worked out in our laboratory. The transection of the sciatic nerve in the hip of rodents is one of the important experimental models of neurotrauma [55,56]. The popularity of this model is associated with the availability of the sciatic nerve in the mid-thigh of the animal for surgical dissection and the decreased inconvenience and stress of the animal in comparison with axotomy of the nerves in the upper extremities [34,36,56].

The rat dorsal root ganglia (DRG) are composed primarily of sensory neurons receiving information from the sciatic nerve that innervates the hind limbs. Each neuron is surrounded by a layer of glial cells. After transection of the sciatic nerve, DRG neurons are axotomized [36,55].

The transection of the right sciatic nerve in the mid-thigh of rats was performed according to the technique described by Savastano et al. [56]. General anesthesia of the animals was carried out by intramuscular injection of 0.75 mL of a mixture of Xyla and Telazol in a 2:1 ratio. Xyla preparation: 2% xylazine hydrochloride solution (Interchemie Werken “de Adelaar” BV, The Netherlands). Telazol: Tiletamine hydrochloride/zolazepam hydrochloride (Zoetis, USA). Symmetrical ganglia from the contralateral side of the same animal were used as a control. The euthanization of animals by decapitation with a guillotine was carried out 1, 4, 24 h, or 7 days after unilateral transection of the right sciatic nerve.

### 4.3. Immunofluorescence Microscopy

After fixation with 4% PFA and washing, rat DRG samples were placed in 20% sucrose solution and embedded in 4% agarose gel (Agarose LM, Dia-m, 1925, 0025). Vibratome 20 μm sections of DRG were washed with PBS and incubated with 5% BSA and 0.3% Triton X-100 to block nonspecific binding sites. The sections were then incubated overnight at 4 °C in the same solution with primary rabbit anti-E2F1 antibodies (SAB2103144, 1:100); mouse anti-NeuN antibody (MAB377; 1:1000) or anti-GFAP antibody (SAB5201104; 1:1000). After washing in PBS, sections were incubated 1 h with the fluorescing secondary antibodies: anti-rabbit CF488A (SAB4600045, 1:1000) or anti-mouse CF555 (SAB4600302, 1:1000). The nuclei of all neurons and glial cells were visualized using Hoechst 33342 (Cat. No. 14533), which stains chromatin [57]. The negative control did not contain any primary antibodies. The sections were mounted on slides in 60% glycerol/PBS. The analysis was performed on 10 images for each of the 21 animals in each group.

To study the expression of E2F1 in the crayfish MRN, we incubated intact or axotomized stretch receptors in van Harreveld’s solution until axon transection, after 4 h, or 8 h. The preparations were fixed with 4% paraformaldehyde, washed, incubated with rabbit anti-E2F1 antibody (SAB2103144, 1:100), then washed and incubated with a secondary anti-rabbit IgG antibody (H + L) labeled with CF™ 488A (SAB4600045; 1:500), washed again and embedded in glycerol. The preparations were also fluorochromated with Hoechst-33342 (Cat. No. 14533). E2F1 levels in different parts of the crayfish stretch receptor, nucleus, and perikaryon (2–4 μm widths with a cytoplasmic ring around the nucleus) of the mechanoreceptor neuron, were analyzed by fluorescence intensity using the ImageJ software (http://rsb.info.nih.gov/ij/ (accessed on 4 October 2021)). The measurement data were normalized to the background fluorescence intensity:

I_norm = (I_mean-I_back)/I_back

where I_mean is the average intensity in the given area (nucleus, perikaryon), and I_back is the average background intensity.

Analysis of the average fluorescence level of the studied protein in the crayfish MRN was carried out using one photograph for each of 10 experimental and 10 control crayfish.

Co-localization of E2F1 with the NeuN neuron marker or glial fibrillary acidic protein (GFAP) was assessed using the ImageJ program (Rasband, W.S., ImageJ, U.S. National Institutes of Health, Bethesda, Maryland, USA, https://imagej.nih.gov/ij/ (accessed on 4 October 2021)) with the JACoP plugin. The co-localization coefficient M1 reflects the proportion of pixels in the green channels (E2F1) relative to the total signal recorded in the red channel (NeuN neuron marker/GFAP) [58,59]. At least 100 cells were used in the calculations. To quantify the mean level of E2F1 fluorescence in experimental and control DRG preparations, 10 control and 10 experimental images were used for each of the 7 rats. The average (by area) fluorescence of the cytoplasm and nucleus for each cell was estimated and the obtained values were averaged. The data obtained on crayfish ganglia were expressed in relative units (rel.un.): the average fluorescence (by area) of the nucleus and perikaryon was estimated, the background was subtracted, and divided by the background (the point most distant from the object).

Crayfish stretch receptor preparations and rat DRG samples were photographed using an Olympus BX-51 fluorescent microscope equipped with an OrcaFlash 4.0 V3 digital camera (Hamamatsu, Japan) at approximately 535 nm excitation wavelengths for Anti-Mouse IgG1 (γ1) labeled with CF555, 488 nm for anti-rabbit IgG (H + L) labeled with CF488A and 365 nm for Hoechst-33342. Fluorescence was recorded at wavelengths >580 nm and >460 nm, respectively.

### 4.4. Visualization of Rat DRG Apoptotic Cells (TUNEL Assay)

Visualization of apoptotic cells was performed using the TUNEL assay (Terminal deoxynucleotidyltransferase (TdT)-mediated dUTP nick end labeling), which marks DNA strand breaks using the In Situ Cell Death Detection Kit, TMR red (#12156792910, Roche). For this purpose, the isolated ganglia 7 days after axotomy were fixed for 6 h in 4% paraformaldehyde and incubated for 48 h in 20% sucrose at 4 °C. Then, they were placed in a 4% agarose gel (Agarose LM, Dia-m, 1925, 0025). The gel blocks were cut on a Leica VT 1000 S vibratome (Nussloch, Germany). To detect apoptosis of rat DRG cells, an In Situ Cell Death Detection Kit, TMR red (#12156792910, Roche) was used. Sections were first incubated at 37 °C with a primary antibody against E2F1 (SAB2103144, 1:100) (green fluorescence), then washed, treated with reagents from this kit, and incubated for 1 h with a secondary antibody Anti-Rabbit CF488A (SAB4600045, 1:1000) (red fluorescence) and with the marker of cell nuclei Hoechst 33342 (10 μg/mL, blue fluorescence) and incubated for 1 h at 37 °C.

The apoptotic index (AI) was calculated for TUNEL-positive cells at a magnification of 20× using the formula: AI = Number of TUNEL-positive cells/Total number of cells (stained with Hoechst 33342) × 100.

Additionally, the co-localization of E2F1 (green signal) with the apoptosis marker (red signal) was assessed using the ImageJ software (http://rsb.info.nih.gov/ij/) with the JACoP plugin. On the image, the Manders coefficient M1 was calculated, which reflects the fraction of pixels with a red signal containing a green signal to the total signal from the red channel. The preparations were examined using an Olympus BX51WI fluorescence microscope equipped with an ORCA-Flash4.0 V3 digital camera (Hamamatsu, Iwata, Japan). The fluorescence excitation range was 570–620 nm. The TUNEL-positive cells were counted in 3 sections of the 5th right (injured) and left (control) DRG obtained from 7 animals at 7 days after sciatic nerve transection. Values obtained were expressed as mean ± S.E.M, n = 7.

### 4.5. Western Blotting

For Western blotting at 1, 4, 24 h, or 7 days after axotomy, the 4th and 5th DRG of 3 rats or the 6 VNC ganglia of 5 crayfish were combined. The protocol for the isolation of cytoplasmic and nuclear fractions was the same for rat DRG and VNC ganglia.

Cytoplasmic and nuclear fractions were obtained using the CelLytic™ NuCLEAR™ Extraction Kit (NXTRACT, Sigma-Aldrich, St. Louis, MO, USA). To do this, the samples were homogenized on ice for 3 min using a Vibra-Cell VCX 130 ultrasonic homogenizer (Sonics, Newtown, CT, USA) in Lysis Buffer, which is included in the CelLytic™ NuCLEAR™ Extraction Kit, supplemented with a mixture of inhibitors of proteases and phosphatases (PPC1010, Sigma-Aldrich) necessary for the preservation of proteins and their phosphorylated forms, as well as nuclease benzonase (E1014, Sigma-Aldrich), which degrades nucleic acids. After homogenization, the samples were centrifuged for 20 min at 10,000–11,000× *g* at 4 °C in a Mikro 220R centrifuge (Hettich, Kirchlengern, Germany). Then, the supernatant containing cytoplasmic proteins was collected, and nuclear proteins were extracted from the sediment containing cell fragments and cell nuclei using the Nuclear Extraction Buffer included in the NXTRACT reagent kit. To do this, the pellet was resuspended and incubated for 40 min with this buffer. After that, the lysate was centrifuged for 5 min at 20,000–21,000× *g* at 4 °C. In protein extracts, the content of total protein was determined using the Bradford reagent (B6916, Sigma-Aldrich, St. Louis, MO, USA).

Acetylated histone H4 (ac-H4) protein was used as a nuclear fraction marker. We used Anti-acetyl-Histone H4 produced in rabbits (#06-866, Merck) at a dilution of 1:500 (Supplementary Figure S1a). The protein glyceraldehyde-3-phosphate dehydrogenase (GAPDH) was used as a marker of the cytoplasmic fraction. We used the Anti-GAPDH antibody produced in rabbits (G9545, Sigma-Aldrich) at a dilution of 1:1000 (Supplementary Figure S1b).

Samples were separated by polyacrylamide gel electrophoresis followed by electrotransfer to a polyvinyl difluoride membrane using the Trans-Blot^®^ Turbo Transfer System (Bio-Rad). After washing and incubating in blocking buffer TBS, the membrane was incubated with primary rabbit anti-E2F1 antibody (SAB2103144; 1:1000), mouse anti-β-actin antibody (A5441; 1:5000), rabbit anti-caspase 3 active (cleaved) form (AB3623, 1:500) and rabbit anti-p53 (P5813, 1:400). After washing in TTBS buffer, the membrane was incubated with secondary antibodies Anti-Rabbit IgG-Peroxidase (A6154; 1:1000), and then with Clarity Western ECL. β-actin contributes to 10–20% of the total cellular protein and is considered to be evolutionarily conserved. Thus, results were normalized to the β-actin, and immunoblots were developed in the Fusion SL gel documentation system (Vilber Lourmat) using VisionCapt software (Vilber Lourmat, France, https://visioncapt.software.informer.com/ (accessed on 4 October 2021)). The gray values of targeted bands were analyzed using the Vision Capt software package. The obtained results were expressed as relative units (rel.un.): the ratio of the optical density of the strip of the protein studied to the optical density of the strip of the protein load marker (β-actin). Negative control: without primary antibodies.

### 4.6. Pharmacological Inhibition of E2F1 In Vivo

We examined the effect of pharmacological inhibition of E2F activity in a model of dorsal root ganglia of rats with transected sciatic nerves (SNT) to assess whether this transcription factor contributes to axotomy-induced apoptosis of DRG cells. For this, a pan E2F inhibitor, HLM006474, also listed in CAS 353519-63-8 (324461, Sigma-Aldrich) was used. It is a small-molecule E2F inhibitor that blocks the transcriptional activity of E2F by disrupting E2F-DNA binding [55,56].

After anesthesia by intramuscular injection of 0.75 mL of xylazine and telazole (2:1) and sciatic nerve transection, the wound was sutured. E2F chemical inhibitor HLM006474, abbreviated here to 6474 (Sigma-Aldrich) was administered intraperitoneally (100 mg/kg) immediately after axotomy and daily during the following 7 days (axotomy group + 6474). The dose of 6474 was selected based on previous in vivo studies of our colleagues [45,46,47] and pilot experiments in our laboratory. The number of animals was n = 21. The animals of the control group were injected with DMSO according to the same scheme. Animals were decapitated 7 days after sciatic nerve transection. After decapitation, the 4th and 5th DRGs were removed bilaterally, frozen in liquid nitrogen, and stored at −80 °C. Furthermore, the level of apoptosis in the obtained samples was determined by the TUNEL method described above, and the levels of proapoptotic proteins, active caspase 3 and p53, were determined by immunoblotting.

### 4.7. Statistical Analysis

Data are presented in this manuscript as mean ± standard error of mean (SEM) from seven or more independent experiments as indicated by n, where “n” refers to biological replicates. Statistical analyses included mean, standard error, confidence interval, limits, linear regression, One-way ANOVA for multiple group comparison, Two-way ANOVA for multiple groups with two independent variables, and a *t*-test for grouped or paired data analysis of variance. Immunoblotting and immunofluorescence microscopy data from rat samples (data presented in Figure 1 and Figure 3) were analyzed by Two-way ANOVA with Dunnett’s a posteriori test (we compared three time points and compared contralateral versus ipsilateral ganglion). One-Way ANOVA with Dunnett’s a posteriori test was used to statistically process data from crayfish experiments (immunoblotting and immunocytochemistry data presented in Figure 2 and Figure 6), where we compared the values of the three experimental groups (time after axotomy). The effect of E2F1 inhibition on the level of pro-apoptotic p53 proteins and active caspase 3 in a rat model of axotomy was assessed by comparing the contralateral vs. ipsilateral ganglion and comparing the DMSO-treated group with the inhibitor-treated group. For this, a Two-way ANOVA with Dunnett’s a posteriori test was used (immunoblotting, Figure 8). The student’s *t*-test was used to assess the level of apoptosis in the axotomized ganglia and nerve of rats compared to non-axotomized samples (Figure 5b), as well as to assess the level of apoptosis under the action of an E2F1 inhibitor compared to the control group (Figure 7). E2F1 colocalization with an apoptosis marker (Figure 5c) and with a glial cell marker (Figure 4) was also assessed by comparing the mean values in two samples using a *t*-test. We performed statistical analysis using SigmaPlot 12.5 statistical software. The post-hoc analysis approach used for ANOVA analysis is Dunnett’s test. A *p*-value of ≤0.05 was considered significant.

## 5. Conclusions

The main conclusions from the research are:

1. Immunoblotting and immunofluorescence microscopy revealed increased expression of E2F1 as early as 4 h and even 1 h after axotomy of mechanoreceptor neurons and ganglia of crayfish ventral nerve cord, as well as rat dorsal rat ganglia;
2. E2F1 is directly involved only in the initiation of apoptosis (4 and 24 h after axotomy);
3. 7 days after transection of the sciatic nerve, the expression of E2F1 is low;
4. The level of E2F1 expression increased both in the cytoplasm and the nuclei of post-axotomy neurons;
5. E2F1 was localized both in axotomized rat dorsal ganglia neurons and glial cells;
6. Increased expression of E2F1 in axotomized ganglia is associated with increased protein expression in neurons, but not in dorsal rat ganglia glial cells;
7. Pharmacological inhibition of E2F in vivo has a pronounced neuroprotective effect: it completely abolishes axotomy-induced increased expression of proapoptotic active caspase 3 and p53 proteins, and also protects axotomized DRG cells from apoptosis.

## Data Availability

Not applicable.

## Supplementary Materials

The following supporting information can be downloaded at: https://www.mdpi.com/article/10.3390/ijms23084451/s1.
