# Supplementary material for: E2F1 Expression and Apoptosis Initiation in Crayfish and Rat Peripheral Neurons and Glial Cells after Axonal Injury
Source: Int J Mol Sci. 2022 Apr 18;23(8):4451. doi: 10.3390/ijms23084451 (PMC9026502; doi:10.3390/ijms23084451)
Supplement: Supplementary file 1 [file ijms-23-04451-s001.zip › ijms-1671627-supplementary.pdf]

Supplementary Materials:

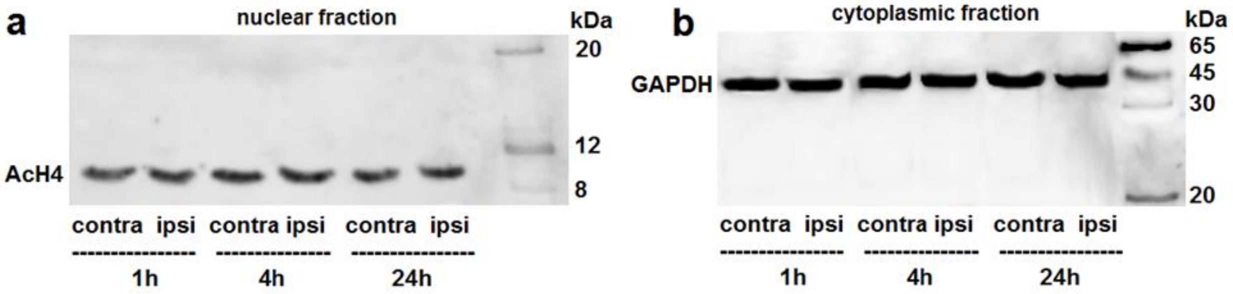

Figure S1. Evaluation of the purity of the obtained nuclear and cytoplasmic fractions by Western blotting of DRG in 1, 4, and 24 h after sciatic nerve transection (axotomy) in rats in comparison with con-tralateral ganglia of the same animals. (a) Acetylated histone H4 (ac-H4) protein was used as a nuclear fraction marker. We used Anti-acetyl-Histone H4 produced in rabbits (# 06-866, Merck) at a dilution of 1:500. (b) The protein glyceraldehyde-3-phosphate dehydrogenase (GAPDH) was used as a marker of the cytoplasmic fraction. We used the Anti-GAPDH antibody produced in rabbits (G9545, Sigma-Aldrich) at a dilution of 1:1000. Denotation: ipsi—axotomized ipsilateral ganglion, contra—contralateral control ganglion.
